# Atrial Dyssynchrony Measured by Strain Echocardiography as a Marker of Proarrhythmic Remodeling and Oxidative Stress in Cardiac Surgery Patients

**DOI:** 10.1155/2020/8895078

**Published:** 2020-12-30

**Authors:** Francisco J. Sánchez, Valeria A. Gonzalez, Martin Farrando, Ariel O. Baigorria Jayat, Margarita Segovia-Roldan, Laura García-Mendívil, Laura Ordovás, Natalia J. Prado, Esther Pueyo, Emiliano R. Diez

**Affiliations:** ^1^Department of Morphophysiology, School of Medicine, National University of Cuyo, Centro Universitario, Mendoza 5500, Argentina; ^2^Department of Cardiovascular Surgery, Clinic of Cuyo, Mendoza 5500, Argentina; ^3^Department of Cardiology, Lagomaggiore Hospital, Mendoza 5500, Argentina; ^4^Biomedical Signal Interpretation and Computational Simulation (BSICoS), Aragon Institute of Engineering Research (I3A), University of Zaragoza Instituto de Investigación Sanitaria (IIS), Zaragoza 50018, Spain; ^5^Aragon Agency for Research and Development (ARAID), Zaragoza 50018, Spain; ^6^Institute of Experimental Medicine and Biology of Cuyo (IMBECU)-CONICET, Mendoza 5500, Argentina; ^7^Biomedical Research Networking Center in Bioengineering, Biomaterials and Nanomedicine (CIBER-BBN), Zaragoza 50018, Spain

## Abstract

Aging leads to structural and electrophysiological changes that increase the risk of postoperative atrial arrhythmias; however, noninvasive preoperative markers of atrial proarrhythmic conditions are still needed. This study is aimed at assessing whether interatrial dyssynchrony determined using two-dimensional speckle tracking echocardiography relates to proarrhythmic structural and functional remodeling. A cohort of 45 patients in sinus rhythm referred for cardiac surgery was evaluated by echocardiography and surface electrocardiogram the day before the intervention. Transmembrane potential, connexin, and potassium channel distribution, inflammatory, and nitrooxidative markers were measured from right atrial tissue obtained from patients. A difference greater than 40 milliseconds between right and left atrial free wall contraction confirmed the presence of interatrial dyssynchrony in 21 patients. No difference in relation with age, previous diseases, and 2-dimensional echocardiographic findings as well as average values of global longitudinal right and left atrial strain were found between synchronic and dyssynchronic patients. Postoperative atrial fibrillation incidence increased from 8.3% in the synchronic group to 33.3% in the dyssynchronic ones. P wave duration showed no difference between groups. Action potentials from dyssynchronous patients decreased in amplitude, maximal rate of depolarization, and hyperpolarized. Duration at 30% of repolarization increased, being markedly shorter at 90% of repolarization. Only the dyssynchronous group showed early and delayed afterdepolarizations. Atrial tissue of dyssynchronous patients displayed lateralization of connexin 40 and increased connexin 43 expression and accumulation of tumor necrosis factor-*α* in the intercalated disc. Tumor necrosis factor-*α* did not colocalize, however, with lateralized connexin 40. Nitroxidative marks and K_ATP_ channels increased perivascularly and in myocytes. Our results demonstrate that, as compared to a traditional surface electrocardiogram, the novel noninvasive echocardiographic evaluation of interatrial dyssynchrony provides a better identification of nonaged-related proarrhythmic atrial remodeling with increased susceptibility to postoperative atrial fibrillation.

## 1. Introduction

Arrhythmias usually complicate cardiovascular surgery, and aging is the main risk factor. Atrial fibrillation is the most frequent sustained arrhythmia with a peak of appearance between the second and fifth days of the postoperative stay [1]. Its incidence progressively increases from 18% in sexagenarians to 50% in octogenarians [2]. Arrhythmic events are usually self-limited, and treatment frequently restores sinus rhythm. However, postoperative atrial fibrillation (POAF) prolongs hospital stay and increases the risk of stroke and mortality [1].

Atrial aging is an elusive prooxidative and proarrhythmic condition. Oxidative stress, hyperadrenergic states, and inflammation contribute to age-related atrial remodeling, but these tissue alterations are challenging to identify noninvasively [3, 4]. In this context, atrial enlargement, stiffness, and conduction blockade are known risk factors, but they are present in only a few patients. Therefore, the mechanisms involved in the onset and perpetuation of POAF are difficult to foresee [5]. Additionally, surgery *per se* facilitates arrhythmias due to ischemia-reperfusion injury, which increases the preexisting oxidative stress state. The lack of tools to estimate the proarrhythmic substrate evidences the absence of preventive interventions.

Structure and function of the beating heart can reveal atrial hidden oxidation and inflammation. Signs of tissue remodeling, before dilatation, arise from atrial dynamic cyclic changes. New echocardiographic techniques like strain and strain rate represent the magnitude and rate of myocardial deformation. Strain can reflect distensibility and atrial contractility [6]. Nonmyocytic cells and extracellular matrix mainly affect distensibility [7]. Cardiomyocyte structure and intercellular communication determine contractile function. Each atrial segment follows a trajectory during the cardiac cycle representative of the tissue physiology [8]. Electrical remodeling involves alterations in both myocytes and nonmyocytic cells, which manifest as contractile dyssynchrony in the echocardiogram, in a similar way as reported for the ventricles [9].

Gap junctions in the heart provide low resistance pathways for propagating the action potential across the myocardium, contributing to electrical coupling and signal propagation [10]. Alteration of cardiomyocyte gap junctions and their main components, connexins (Cx), has been suggested to contribute to the formation of arrhythmias, including atrial fibrillation [11–13]. Accumulating evidence also suggests that inflammation and oxidative stress are involved in atrial remodeling. Detection of protein 3-nitrotyrosine is regarded as a marker of nitrooxidative stress and is observed especially in inflammatory processes. The reaction of peroxynitrite with tyrosine leads to the formation of 3-nitrotyrosine and promotes protein, lipid, and DNA damage [14, 15]. ATP-regulated potassium channels (K_ATP_) are well-characterized metabolic and oxidative sensors in ischemia/reperfusion arrhythmias and here postulated as an interesting substrate of POAF [16].

This study is aimed at assessing whether interatrial dyssynchrony, determined by using two-dimensional speckle tracking echocardiography, relates to proarrhythmic structural and functional remodeling of the atria and whether this increases the susceptibility for POAF.

## 2. Materials and Methods

### 2.1. Subjects and Ethical Considerations

Patients with coronary artery disease, aortic stenosis, or the combination of both pathologies, scheduled for surgery at the Department of Cardiac Surgery (Clinic of Cuyo, Mendoza, Argentina), were prospectively enrolled between January 2018 and March 2020. Coronary disease and aortic valve stenosis severity were defined according to current ESC Guidelines to determine surgery indication [17, 18]. All subjects provided written informed consent under the research protocol approved by the Ethics Committee of the National University of Cuyo (Exp-Cuy: 22959/2017).

Clinical data including age, gender, and history of previous myocardial infarction and heart failure was collected. The presence of preoperative atrial fibrillation was determined according to previous electrocardiographic reports or diagnosis in medical history. Information regarding the following cardiovascular risk factors was collected: hypertension, dyslipidemia (low-density lipoprotein cholesterol above 100 mg/dL or the use of lipid-lowering drugs), smoking (current or any smoking habit in the past ten years), and diabetes mellitus (previous diagnosis of diabetes mellitus or glycated hemoglobin greater than 6.5%). The preoperative use of medications was also documented.

### 2.2. Inclusion and Exclusion Criteria

Patients over 18 years of age in sinus rhythm with an indication of cardiovascular surgery that gave written informed consent were included in the study. Exclusion criteria were as follows: indication of mitral or tricuspid valve repair or replacement, being older than 80 years, history of previous atrial fibrillation, presence of moderate valvular disease or valvular prosthesis, history of congenital cardiac abnormalities or cardiac tumors, emergency surgery, inability to provide informed consent, and a not entirely detectable left and right atrial profile from the apical four-chamber view during preoperative echocardiography.

### 2.3. Preoperative Electrocardiogram

Before surgery, patients underwent a 12-lead electrocardiogram (ECG) using the Synchronous ECG software V1.3.5. Measurement in milliseconds of the P wave and the PR segment was performed with the software caliper. According to the current classification, these patients were evaluated for the presence of an interatrial conduction disturbance called Bayes syndrome [19]. There are two major categories for this syndrome: complete and incomplete, both based on P wave duration and morphology in the 12-lead ECG.

### 2.4. Echocardiography and Atrial Strain

All patients were imaged in a left lateral decubitus position using ESAOTE ultrasound system equipment (MyLab30Gold Cardiovascular) with a 2-4 MHz/PA240 probe. Two-dimensional speckle tracking strain imaging was performed from the apical position by an experienced technician. The average frame rate for analysis was 60-80 frames/s. During a single breath-hold, three consecutive cardiac cycles were stored digitally for off-line analysis in the four-, two-, and three-chamber view. The entire right and left atriums were carefully visualized to prevent walls' dropout.

Measurements focused on evaluating the indexed volume of the left atrium and the area of the right atrium. In the parasternal long-axis or short-axis view, M-mode of the left ventricle chamber was measured for diameter and wall thickness. In the apical 4-chamber view, the left ventricle ejection fraction was determined using the Simpson measurement. Mitral and tricuspid inflows were recorded at the tip of the valve leaflets. The peak velocities of early and late diastolic filling waves (E wave and A wave) and the E/A velocity ratio were measured. The e velocity was obtained by tissue Doppler averaging the lateral and septal mitral annulus values and the e wave of the tricuspid lateral wall. E/e' values were obtained from both ventricles.

**Figure 1:**
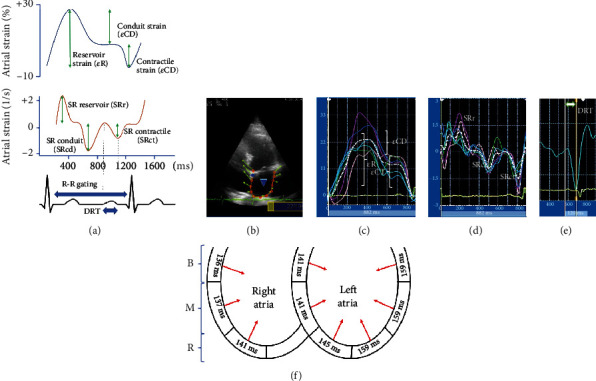
Two-dimensional atrial strain curves. (a) Atrial strain and strain rate curves and determination of atrial mechanics according to an R-R wave-timed analysis. Deformation rate time (DRT) is determined from the beginning of the P wave in the electrocardiogram to the maximum deflection of the strain rate contractile phase (STct). (b) Four-chamber view of speckle tracking left atrial strain. (c) Atrial strain curves. The white, dashed, and dotted line shows the average strain curve for the six segments of the atria in that view. (d) Atrial strain rate curves. (e) Determination of DRT in a segment of left atria. The measurement agreed with the scheme in (a). (f) Schematic four-chamber view of how DRT can show the activation in time of different segments from both atria. B: base segment; M: medium segment; R: roof segment.

Strain and strain rate datasets were analyzed using a wall motion tracking software (ESAOTE MyLab). In apical views (4-chamber, 3-chamber, and 2-chamber), left atrial endocardial boundaries were manually measured at the end-diastolic phase. Right atria were only registered in the 4-chamber view. The values of the reservoir, conduit, and atrial contraction strain were recorded according to the EACVI/ASE/Industry Task Force to standardize deformation imaging [20]. The values of strain rate for the reservoir phase, the conduit, and contraction phases were also recorded (Figure 1). The same was also done in 4 chambers for the right atrium. The left atrium was subsequently divided into basal, medial, and roof atrial segments. This determined a total of 15 segments for the left atria when the 3 apical views were added. The right atrium was evaluated similarly, but only the lateral segments were taken into account, adding 3 more segments, thus making a total of 18 segments. Septal segments were considered as left atria.

Deformation rate time (DRT) was recorded from the beginning of the P wave in the ECG to the maximum deflection of the atrial contraction in the strain rate for each of the segments, as indicated in Figures 1(a) and 1(e).

**Figure 2:**
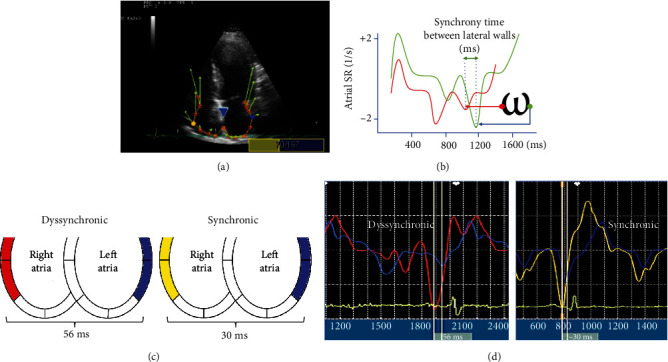
(a) Longitudinal strain echocardiography in a four-chamber view with the Omega (*ω*) adaptation for the analysis technique of time activation between lateral walls. Yellow and blue dots show the measured segment of the lateral wall. (b) Measurement technique to determine differences between the activation time of two segments at the lateral atrial walls, from one maximal deflection strain rate contraction of a curve to the same point in the other curve. (c) Schematic visualization in a four-chamber view with an example of the determination of time difference in activation between the lateral wall of the right and left atria. (d) Example of strain rate from right and left atrial walls in synchronic and dyssynchronic patients.

To determine interatrial synchrony, an adaptation of atrial strain was performed to evaluate the right and left atrial walls at the same time. This new evaluation was called “Omega” (*ω*) because of the form the strain takes in the images. Here, the maximal time difference between the strain rate contraction peaks of both right and left atrial lateral walls was recorded (Figure 2). Blinded analysis of the echocardiograms was performed by two independent researchers (FJS and VAG).

### 2.5. Atrial Transmembrane Potential

Right atrium samples taken in the operating room were transferred within 15 to 20 minutes to the laboratory using a cold oxygenated Ringer-type solution with albumin. Upon arrival, all samples were dissected into smaller pieces, pinned at the bottom of a perfusion chamber with the endocardial surface facing up, and continuously superfused with a modified Krebs–Henseleit solution containing (in mM): 121 NaCl, 25 NaHCO_3_, 1.2 Na_2_HPO_4_, 5 KCl, 2 CaCl_2_, 1.2 MgSO_4_, and 11 glucose. Once equilibrated with 5% CO_2_ in O_2_ at 36.5 ± 0.5°C, the pH of the solution was 7.4 ± 0.02.

The membrane potential was recorded with flexibly mounted glass microelectrodes from subendocardial trabecular atrial cells. Microelectrodes were filled with 3 mM KCl and had resistances of 10–15 M*Ω*. After 20 to 30 minutes of stabilization, we continuously obtained epicardial transmembrane potential using a custom-made microelectrode amplifier. The signals were digitized with an analog-to-digital converter (NI PCI-6221; National Instruments, Austin, Texas) and recorded using LabView SignalExpress 2.5 (National Instruments, Austin, Texas).

The following properties of the transmembrane potentials were quantified: action potential amplitude, resting potential, maximum upstroke velocity (*Δ*V/Δ*t*_max_), and action potential duration at 30 and 90% of repolarization. Arrhythmic events and arrhythmogenic triggers were evaluated by visual supervision of the traces blindly regarding the patient's characteristics.

### 2.6. Structural, Inflammatory, and Nitrooxidative Evaluation by Fluorescent Immunohistochemistry

Part of each atrial sample was fixed in a 4% paraformaldehyde solution for 1 h at 4°C before embedding in paraffin blocks. Five-micrometer-thick tissue sections were stained using the following primary antibodies or label: rabbit polyclonal anti-Cx40 (Cx40 H-116, Santa Cruz, sc-28658, dilution 1 : 300), rabbit polyclonal anti-Cx43 (Cx43, Abcam, ab11370, dilution 1 : 1000), rabbit polyclonal anti-Kir6.1 (Kir6.1, Thermo Fisher, PA5-48354, dilution 1 : 500), mouse monoclonal anti-TNF*α* (TNF*α* 52B83, Santa Cruz, sc-52746, dilution 1 : 300), mouse monoclonal antinitrotyrosine (3-nitrotyrosine, Santa Cruz, sc-32757, dilution 1 : 200), and mouse monoclonal anti-SERCA2a (ab2817 Abcam, dilution 1 : 1000). Wheat germ agglutinin (WGA) conjugated to Alexa Fluor 555 against the extracellular matrix (W32464 Thermo Fisher, dilution 1 : 500) and fluorescently conjugated F-actin against the intracellular content (ab112124 Abcam, dilution 1 : 300). The secondary antibodies were anti-rabbit conjugated with Alexa Fluor 633 and anti-mouse labeled with the Alexa Fluor 488 (Jackson ImmunoResearch Laboratories Inc., West Grove, PA, USA, dilution 1 : 500). Images were acquired with a confocal microscope Zeiss LSM 880 and processed with the Zen Blue 2.5 software (Carl Zeiss Microscopy GmbH, 2018).

The maximum intensity projection of 20 to 40 z stacks was used to analyze the lateralization of connexins and the integrated optical density (IOD). Lateralization was measured with an open access automated program designed by our group called MARTA [21]. This software generates cell masks, contours individual cells, and splits the cells into 4 rectangles to estimate the lateral-to-total ratio of connexins. The IOD quantified the area (>3 pixels connected) multiplied by the average intensity using the software ImageProPlus 4.5, 2001 (Media Cybernetics, Inc., Rockville, MD, USA). Values of IOD measured for 18 pictures per immunofluorescence channel were grouped after the blinded analysis was completed by two independent researchers (NJP and ERD), and the results were assigned to the corresponding groups. The values of IOD are relatively expressed to the level measured in the synchronic group.

### 2.7. Postoperative Atrial Fibrillation Detection

Continuous ECG monitoring after surgery was performed in the cardiovascular intensive care unit for 48–96 hours to detect any new onset of atrial fibrillation. Arrhythmic events of at least 1 min length were considered POAF when assessed by a monitoring system or using a 12-lead ECG in case of a symptomatic episode that required intervention.

### 2.8. Statistical Analysis

Qualitative variables are expressed as number and percentage. Quantitative variables are expressed as mean ± standard deviation (SD) if they are normally distributed and median with a range if they are not normally distributed. For qualitative data, the chi-square test is used. For nonnormally distributed data, the Mann–Whitney tests are used; for normally distributed data, the Student *t*-test is used. The receiver-operating characteristic (ROC) curve analysis is performed to determine the cutoff value of atrial variables as predictors of POAF. The area under the curve and 95% confidence interval (CI) are used to determine the parameters' incremental diagnostic value. Log-rank (Mantel Cox) test is used to assess the incidence of POAF. A *p* value below 0.05 was chosen as an indicative of statistical significance. The GraphPad Prism version 9.0.0, 2020 (GraphPad Software, San Diego, CA, USA, http://www.graphpad.com) was used for statistical analysis.

## 3. Results

### 3.1. Postoperative Atrial Fibrillation, Patient Characteristics, and Classification regarding Interatrial Synchrony

A total of 45 patients were included in the study. Forty-one underwent coronary artery bypass surgery, three aortic valve replacement, and one combined surgery. The mean age was 67.4 ± 7.7 years, and 69% were male. No patient was under treatment with digitalis or antiarrhythmics.

**Figure 3:**
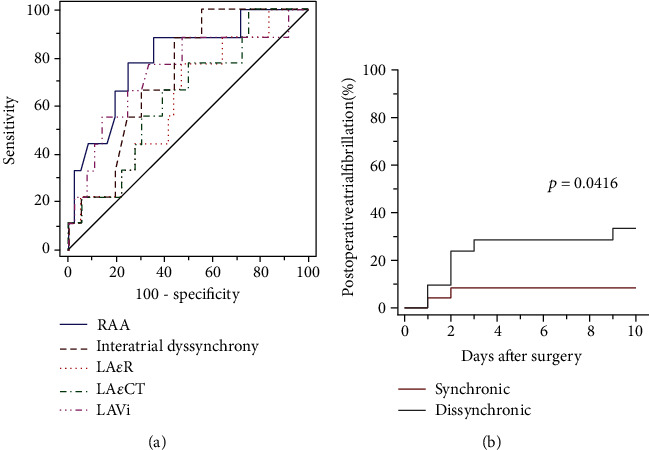
Receiver-operating characteristic curves (ROC) for atrial fibrillation incidence after cardiac surgery. (a) ROC curves for right atrial area (RAA), interatrial dyssynchrony, left atrial reservoir strain (LA*ε*R), left atria contraction strain (LA*ε*CT), and left atrial volume index (LAVi). (b) Postoperative atrial fibrillation incidence in patients with synchronic and dyssynchronic activation of the right and left atrial lateral walls.

This population was divided into synchronic and dyssynchronic patients based on the difference between the activation of the lateral right and left atrial walls determined from the echocardiogram (Omega). This was used as a factor related to the incidence of POAF. A time difference greater than 40 milliseconds had a sensitivity of 88.9% and a specificity of 55.6% for identifying the atrial fibrillation events, with an area under the curve of 0.728 (95% CI 0.575-0.850) (Figure 3(a)).

Dyssynchronic patients suffered from a higher incidence of POAF. The median time of atrial fibrillation onset after surgery was 2.5 days (Figure 3(b)). All patients recovered sinus rhythm prior to discharge from hospital with the use of intravenous amiodarone. No electrical cardioversion was required.

The receiver-operating characteristic curve (ROC) of atrial echocardiographic measurements had the following areas under the curve and cutoff values: right atrial area 0.796 (95% CI 0.650-0.902), cutoff 17.6 cm^2^; left atrial volume index 0.742 (95% CI 0.590-0.861), cutoff 29.86 mL/m^2^; left atria contraction strain 0.645 (95% CI 0.488-0.782), cutoff 15.4%; left atrial reservoir strain 0.636 (95% CI 0.479-0.774), cutoff 30.7%. After adjusting the right atrial area, left atrial volume index, left atrial reservoir strain, and left atria contraction strain for the cutoff values, according to current guidelines, only left atrial volume index remained as predictors of postoperative atrial fibrillation (area under the curve: 0.681 95% CI 0.525-0.812), but the area under the curve was lower than the one obtained with the interatrial dyssynchrony [22–24]. These results supported dyssynchrony as the grouping variable for the comparisons throughout the study.

Patients in the synchronic and dyssynchronic groups did not show differences in age, type of surgery, comorbidity, risk factors, or treatment received before surgery (Table 1). The average pump time of the patients was 118 ± 39 minutes, and the clamping time was 75 ± 24 minutes, with no significant differences between groups. During the study, there was only one death from a patient in the synchronic group.

### 3.2. Atrial-Related ECG

P wave duration in the ECG was 125 ± 15 ms and 118 ± 12 ms in the synchronic and dyssynchronic groups, respectively (*p* = 0.128 by *t*-test). PR interval was longer in the synchronic group than in the dyssynchronic group (179 ± 32 ms vs. 160 ± 25 ms, respectively; *p* = 0.044 by Mann–Whitney test). No significant differences in Bayes syndrome incidence were found between groups (*p* = 0.985).

### 3.3. Echocardiography and Atrial Strain

Regarding general data from the echocardiograms, the mean ejection fraction of the left ventricle was 56.83 ± 15.36%. The mean indexed volume of the left atrium was 30.78 ± 9.04 mL/m^2^, and the area of the right atrium was 16.20 ± 3.36 cm^2^. The E/e' ratio of the left ventricle showed a value of 8.6 ± 3.5, which indicated end-diastole pressures at the normal upper limit in most patients (Table 2). There was no other significant difference between the groups in echocardiographic findings, except for the left ventricular index mass. The analysis of the atrial speckle tracking strain showed no significant differences between groups in the deformation rate times corresponding to the activation of any of the 18 atrial segments (Table 3).

The rest of the usual strain analysis also showed no differences between groups (Table 4).

### 3.4. Action Potentials and Proarrhythmic Triggers

**Figure 4:**
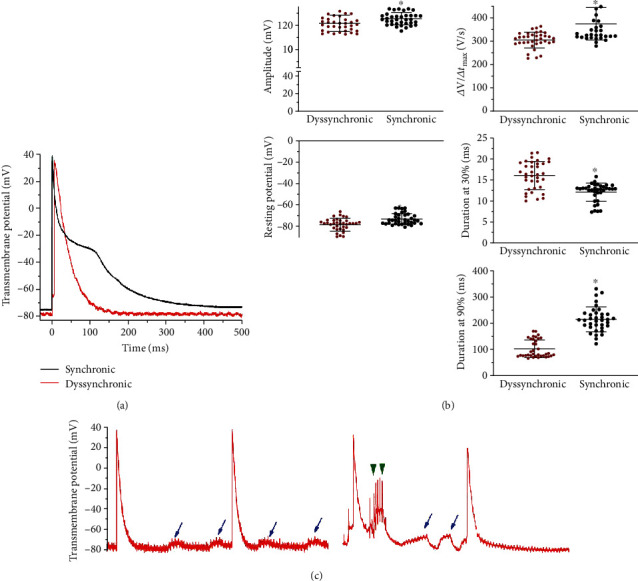
Action potentials and arrhythmic triggers in interatrial dyssynchronic patients. (a) Averaged action potentials from three different patients from each group; (b) quantification of action potential amplitude, resting membrane potential, maximum upstroke velocity (*Δ*V/Δ*t*_max_), and the action potential duration at 30 and 90% of the repolarization from both groups. ^∗^*p* < 0.01 by *t*-test; (c) arrhythmogenic triggers in 2 seconds traces from two dyssynchronic patients. The blue arrows mark delayed afterdepolarizations, which are very frequent in dyssynchronous patients. Green arrowheads indicate triggered events from early afterdepolarizations.

Action potentials from dyssynchronous patients decreased by 3.9 ± 1.9 mV in amplitude and by 66 ± 13 V/s in the maximal upstroke velocity (Figures 4(b) and 4(c)).

During the beginning of the upstroke of the action potential, we observed that a segment of the ascendent activation had a little nudge, like a foot that delayed the maximal upstroke occurrence. The resting membrane potential was hyperpolarized by 3.3 ± 1.3 mV in the atrial tissue samples from dyssynchronic patients. The action potential duration at 30% of repolarization increased by 4.0 ± 0.7 ms but markedly shortened when measured at 90% of repolarization by 107 ± 12 ms. Only action potentials from synchronic patients displayed a plateau and slow repolarization typical of a standard atrial action potential. Early and delayed afterdepolarizations were only observed in the dyssynchronic group.

### 3.5. Atrial Remodeling, Inflammation, and Nitrooxidative Stress

**Figure 5:**
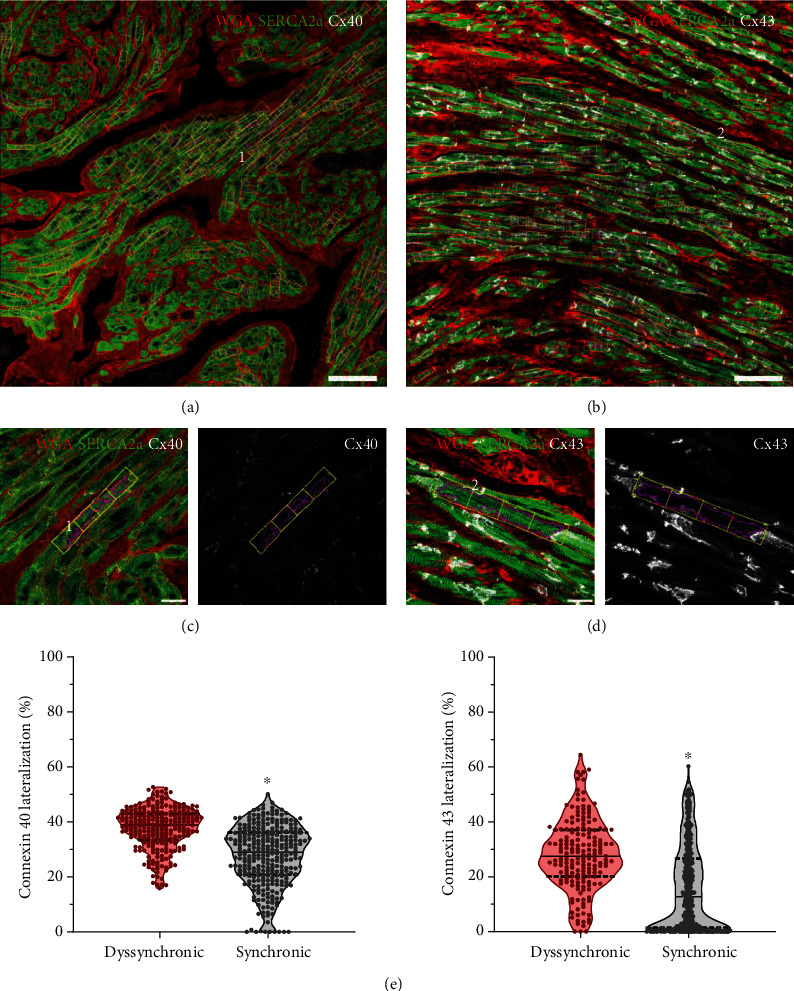
Automatic quantification of Cx40 and Cx43 lateralization. Cardiomyocyte detection in samples marked with wheat germ agglutinin (WGA) and antibodies anti-SERCA2a and anti-Cx40 in (a) or anti-Cx43 in (b). White bars correspond to 100 μm. The numbers 1 and 2 are used to trace the origin of the myocytes shown in (c) and (d). Each cell is divided into 4 rectangles to estimate the lateral-to-total ratio of connexins from the individual channel of the connexins marks. White bars correspond to 20 μm. (e) Median and quartiles of the percentage values for the lateralization of each connexin and ^∗^*p* < 0.01 by Mann–Whitney tests.

Cx40 lateralization increased by 9.81% (95% CI of difference 7.71-10.93) in samples from dyssynchronic patients with respect to synchronic ones (Figure 5).

**Figure 6:**
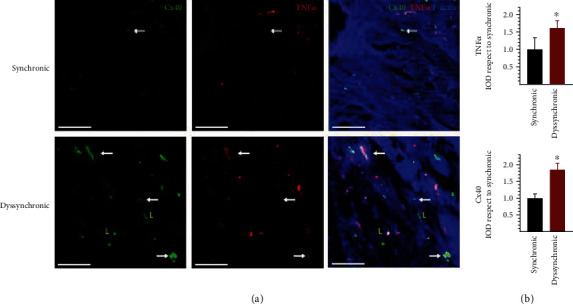
Analysis of Cx40 and TNF*α* localization and expression in atrial samples from synchronic and dyssynchronic patients. (a) White arrows indicate colocalization of both markers, while the yellow L letters indicate lateralized Cx40. F-Actin marks cellular structures. White bars correspond to 50 *μ*m. (b) Quantification of TNF*α* and Cx40 integrated optical density (IOD) displayed as mean and SD and ^∗^*p* < 0.05 by *t*-test.

This lateralization was not colocalized with the increased signal of tumor necrosis factor-*α* (TNF*α*) seen in the dyssynchronic group (Figure 6). The TNF*α* was mainly colocalized with Cx40 close to the intercalated discs.

**Figure 7:**
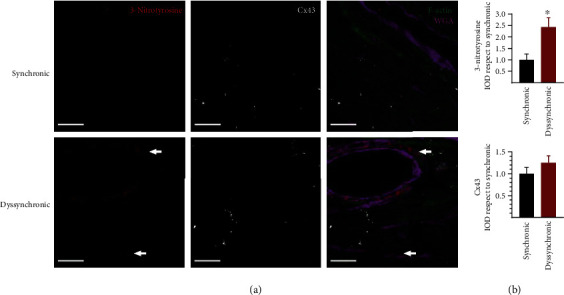
Detection of nitrooxidative stress levels and Cx43 expression in atrial samples from synchronic and dyssynchronic patients. (a) An increase in perivascular (indicated by the white arrows) and myocytic signals of 3-nitrotyrosine occurred in the dyssynchronic group. F-Actin marks the tissue in green, and wheat germ agglutinin (WGA) marks the membranes in purple to improve structural reference in the tissue. White bars correspond to 20 *μ*m. (b) Quantification of 3-nitrotyrosine and Cx43 integrated optical density (IOD) displayed as mean and SD and ^∗^*p* < 0.05 by *t*-test.

Nitrotyrosine is a relatively stable marker of nitroxidative stress that is formed by peroxynitrite interaction with tyrosine. The nitrotyrosine signal was clearly higher in the dyssynchronic group, especially around the blood vessels. However, it was diffusely observed in the tissue and the microvasculature (Figure 7). Cx43 increased in intercalated discs and also lateralized 14.6% (95% CI of difference 10.81-16.56) more in dyssynchronic patients (Figure 5), but both changes were unrelated to nitrotyrosine marks.

**Figure 8:**
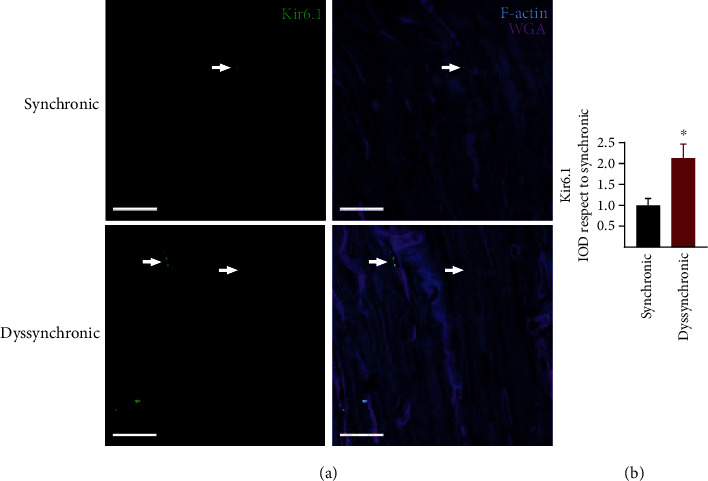
Characterization of K_ATP_ channel expression in atrial samples from synchronic and dyssynchronic patients. (a) The Kir6.1 subunit of the potassium channels was present perivascularly (white arrows) and in atrial cardiomyocytes (arrowheads). F-Actin marks the tissue with a blue staining and WGA cellular membranes to facilitate structural characterization. White bars correspond to 20 *μ*m. (b) Quantification of Kir6.1 integrated optical density (IOD) displayed as mean and SD and ^∗^*p* < 0.01 by *t*-test.

The expression of K_ATP_ channels assessed by the detection of its pore-forming unit Kir6.1 increased in atrial myocytes of dyssynchronic samples (Figure 8). K_ATP_ channels usually aggregated at a vascular level in images of both synchronic and dyssynchronic patients. The action potential shortening and the hyperpolarization observed with the microelectrodes agrees with increased expression of this channel.

## 4. Discussion

Our study found that interatrial dyssynchrony assessed by echocardiography was associated with electrical and structural atrial remodeling and the incidence of postoperative atrial fibrillation. The innovative measurement interatrial dyssynchrony was suitable to estimate noninvasively nitrooxidative and inflammatory substrate, as well as, an electrophysiological arrhythmogenic remodeling.

### 4.1. Atrial Dyssynchrony and Postoperative Atrial Fibrillation

Interatrial dyssynchrony is not a common risk factor for POAF. This is one of the first reports of a preoperative indicator of proarrhythmic tissue remodeling. Reservoir and contractile strain of the left atria have been previously related to the development of paroxysmal and chronic atrial fibrillation [25–27]. In this study, both were below the reference values agreed in the clinical guidelines [22]. This indicates more rigid atria, with less pump function, but without a difference between the two groups. One of the reasons for this absence of difference could be the way in which the patients were classified. We did not separate the groups according to the incidence of atrial fibrillation but to the presence of dyssynchrony determined by echocardiogram. Another reason for our patients having a better pump and reservoir function is that we did not include patients with mitral or tricuspid valve disease known for having larger atrial volumes, more rigid atria, and a clear tendency to develop atrial fibrillation. Also, one of our exclusion criteria was a previous history of atrial fibrillation. We found that interatrial dyssynchrony was a better predictor of postoperative atrial fibrillation than other atrial measurements.

Atrial structural and functional remodeling has many causal factors such as oxidative stress, inflammation, fibrosis, and the normal process of aging. Previous studies have shown that left and right atrial enlargement predicts postoperative atrial fibrillation [28, 29]. All our patients had left atrial size below the cutoff values for atrial enlargement. Moreover, the atrial size was preserved in the dyssynchronic patients despite the higher left ventricle index mass and the slightly reduced conduit strain. Left ventricular hypertrophy is associated with higher end-diastolic left ventricular pressures, explaining the difference in the conduit left atrial strain, but other markers of elevated end-diastolic pressures like E/e' relation showed no differences. Right atrial size had no significant difference between groups but was slightly bigger in the dyssynchronic group.

Evaluating the activation of the different segments of the atria did not show differences between the two groups. This supports the standardization guidelines that recommend the longitudinal analysis of the atria as a whole [22]. Additionally, segment by segment analysis takes a longer time and requires a more experienced operator. Nevertheless, several studies have already shown that intra-atrial dyssynchrony is related to atrial fibrillation development [30, 31]. So, this analysis may be a useful tool for the prediction of arrhythmias. The omega approach, presented here for the first time, is an adaptation of the technique that not only introduces the evaluation of the right atria but it also reduces the time for analysis. This facilitates the interatrial dyssynchrony evaluation with significant potential for arrhythmogenic substrate identification in the preoperative setting.

Structural remodeling produces changes in atrial function. These changes can be accelerated in many disease processes, like hypertension, diabetes mellitus, and ischemic heart disease. These changes can reflect modifications in structural proteins that produce atrial electrical remodeling, which, in turn, could be the cause for longer times of atrial mechanical activation. The results of our study support that atrial dyssynchronic activation is related to both electrical and structural changes, without an apparent influence of other risk factors.

### 4.2. Atrial Dyssynchrony, Action Potentials, Connexins, and K_ATP_ Channels

Dyssynchronic patients presented abnormalities in the upstroke of the action potentials. In multicellular preparations, both Na^+^ channels and connexins contribute to phase 0 of the action potentials. The foot and the delay in the upstroke observed in dyssynchronic patients have been previously associated with connexin alterations (see Figure 3(a)) [32]. Connexin expression, conductance, and location can influence the initial part of the electrical activation [33]. The lateralization of Cx40 and Cx43 shown in Figure 5 could support these findings at the beginning of the action potentials. Still, the concurrent increase in Cx43 at the intercalated discs goes against this idea, but agrees with previous reports that reduce the link with action potential morphology [34]. The reduced amplitude and maximal rate of depolarization indicate an impairment in the sodium currents. Therefore, both sodium channels and connexins can contribute to interatrial dyssynchrony because they are the major determinants of electrical impulse propagation between cardiomyocytes.

K_ATP_ channels could explain the action potential shortening and the hyperpolarization, and these features can worsen under stressful situations like ischemia and reperfusion. Our results bring a new potential substrate to the complex electrophysiology of the atrial tissue. The subunit Kir6.1 is an inward rectifier current mainly expressed in the vasculature [16]. Here, we report the expression in human atrial cardiomyocytes of dyssynchronic patients. The activation of these channels reduces the refractory period, thus facilitating reentrant currents that could lead to postoperative atrial fibrillation. K_ATP_ channels are particularly sensitive to the metabolic and oxidative cellular conditions [35]. In the context of cardiovascular surgery, their properties make them attractive candidates for potential preventive antiarrhythmic therapies.

The afterdepolarizations observed in the dyssynchronic group could be related to alterations in calcium handling and leaks from the sarcoplasmic reticulum through the ryanodine receptors. These alterations have already been described as associated with a more oxidized substrate in patients undergoing coronary artery bypass grafting [36]. Dyssynchrony can promote stretching of the cells during the repolarization phase of shortened action potentials, leading to mechanoelectric feedback capable of early afterdepolarization induction [37].

### 4.3. Atrial Dyssynchrony, Inflammation and Nitrooxidative Stress

The signal with nitrotyrosine was clearly higher in dyssynchronic patients, indicating an increased nitrooxidative state in this group. Accumulating data suggest that inflammation and oxidative stress are involved in the development, recurrence, and persistence of atrial fibrillation [38]. Myocardial oxidative injury can lead to an increased susceptibility to postoperative atrial fibrillation by impairing atrial contraction, altering myofibrillar energetics, and reducing atrial effective refractory period [5]. As a biomarker of oxidative damage, 3-nitrotyrosine has been widely studied. The formation of 3-nitrotyrosine increases in various cardiovascular diseases, such as cardiac failure, hypertension, atherosclerosis, and cardiovascular complication of diabetes [39]. 3-Nitrotyrosine formation associates with atrial fibrillation in patients with mitral valve disease [14, 40]. Peroxynitrite has been suggested to participate in oxidative damage, which may contribute to atrial contractile dysfunction in atrial fibrillation [41]. Here, we found an increase of 3-nitrotyrosine in the dyssynchronic group, making the echocardiogram a potential oxidative stress indicator.

Inflammation levels were clearer in the dyssynchronic group. POAF is associated with a higher expression of NF-*κ*B, myocardial fibrosis, and impaired cardiomyocyte communication before the bypass surgery [11]. The histological results presented here show higher degree of colocalization between TNF*α* patches and the Cx40 close to the intercalated disc. Lateralized Cx40 did not show this association with TNF*α*. The latter weakens the relation between structural remodeling and inflammation. TNF*α* should be interpreted with caution as the signal was disturbed by the fluorescence of red blood cells. An altered substrate of Cx40 can worsen the downregulation described during bypass surgery [42].

## 5. Conclusion

In conclusion, our results demonstrate that a novel noninvasive echocardiographic evaluation of interatrial dyssynchrony is better than the surface ECG to identify proarrhythmic atrial electrical and structural remodeling, with increased susceptibility to POAF. The increase in the K_ATP_ channel and the lateralization of connexins observed in dyssynchronic atria could shorten action potential and predispose hearts to reentrant circuits. Inflammation and nitrooxidative stress can increase the proarrhythmic substrate. These factors were all estimated by a simple, noninvasive technique. Future research using this technique will help to further understand the potential role of atrial dyssynchrony as a preoperative predictor of atrial fibrillation in the context of cardiovascular surgery.

## Figures and Tables

**Table 1:** Demographics and medication of patients before surgery.

Variables	Dyssynchronous (*n* = 21)	Synchronous (*n* = 24)	*p* value
Demographics			
Age in years (*n*)	68 ± 9	67 ± 7	0.262
Patient sex (male)	15 (71.4)	16 (66.6)	0.731
Type of surgery: coronary	20 (95.2)	21 (87.5)	0.559
Coronary disease	20 (95.2)	23 (95.8)	0.923
Diabetes mellitus	13 (61.9)	10 (41.6)	0.175
Hypertension	20 (95.2)	22 (91.6)	0.632
Dyslipidemia	19 (90.5)	20 (83.3)	0.482
Dilated cardiomyopathy	4 (19.0)	1 (4.2)	0.113
Chronic pulmonary obstructive disease	1 (4.8)	3 (12.5)	0.363
Chronic renal failure	3 (14.3)	1 (4.2)	0.234
Obesity	10 (47.6)	8 (33.3)	0.329
Stroke	0	1 (4.2)	0.344
Heart failure in last month	3 (14.3)	5 (20.8)	0.567
Smoker	2 (9.5)	4 (16.6)	0.482
Presurgical medications			
Aspirin use	21 (100)	24 (100)	1
*β* Blocker use	15 (71.4)	17 (70.8)	0.965
Statins	20 (95.2)	24 (100)	0.280
ACEI or ARA	15 (71.5)	14 (58.3)	0.360
Spironolactone or eplerenone	5 (23.8)	2 (8.3)	0.153
Furosemide	3 (14.3)	2 (8.3)	0.526

Data are expressed as number and (%), unless otherwise indicated. *p* values are calculated by Kruskal-Wallis test or chi-square test. ACEI: angiotensin-converting enzyme inhibitors; ARA: angiotensin II, type 1 receptor antagonists.

**Table 2:** Comparative echocardiographic measurements in both groups.

Echocardiographic variables	Dyssynchronous	Synchronous	*p* value
Atrial index volume (mL/m^2^)	32.51 ± 8.27	29.45 ± 9.76	0.534
Right atrial area (cm^2^)	17.07 ± 3.54	15.57 ± 3.13	0.694
Fey Simpson (%)	54.71 ± 15.36	59.21 ± 15.47	0.887
Mitral E wave velocity (cm/sec)	68.14 ± 20.32	71.83 ± 16.82	0.216
Mitral A wave velocity (cm/sec)	85.33 ± 21.44	76.96 ± 18.4	0.568
E/e ratio LV	8.83 ± 3.83	8.53 ± 3.44	0.542
E/e ratio RV	4.42 ± 1.37	4.38 ± 1.08	0.404
LV index mass (g/m^2^)	138.29 ± 52.97	108.33 ± 33.61	0.021

Data are express as mean and SD. *p* values are analyzed by *t*-test. LA: left atria; RA: right atria; LVEF: left ventricular ejection fraction; LV: left ventricle; RV: right ventricle.

**Table 3:** Deformation rate time per segment in both groups.

Deformation rate time	Dyssynchronous	Synchronous	*p* value
Left atria			
Base septum segment	138.95 ± 44.43	142.46 ± 39.48	0.780
Medium septum segment	146.57 ± 40.89	136.54 ± 30.11	0.350
Roof septum segment	157.76 ± 47.46	134.46 ± 30.11	0.078
Base lateral segment	168.38 ± 45.32	150.79 ± 41.49	0.181
Medium lateral segment	169.48 ± 41.77	149.67 ± 42.23	0.122
Roof lateral segment	161.67 ± 33.42	156.83 ± 41.26	0.671
Base anterior segment	151.71 ± 43.26	155.67 ± 34.38	0.734
Medium anterior segment	154.05 ± 41.87	154.54 ± 35.02	0.966
Roof anterior segment	157.05 ± 41.48	151.67 ± 49.59	0.697
Base inferior segment	159.52 ± 48.36	140.04 ± 30.32	0.108
Medium inferior segment	154.24 ± 51.01	144.42 ± 28.44	0.422
Roof inferior segment	161.43 ± 63.04	150.08 ± 33.65	0.447
Base inferior lateral segment	153.29 ± 49.10	145.38 ± 35.49	0.535
Medium inferior lateral segment	150.90 ± 44.43	148.04 ± 31.73	0.803
Roof inferior lateral segment	133.62 ± 45.81	151.54 ± 27.04	0.112
Right atria			
Base lateral segment	138.29 ± 62.95	135.17 ± 42.17	0.844
Medium lateral segment	131.24 ± 57.68	141.71 ± 35.19	0.460
Roof lateral segment	138.81 ± 62.89	143.88 ± 35.68	0.737

Data are expressed as mean and SD. *p* values for analysis by unpaired *t*-test.

**Table 4:** Comparative atrial strain measurements in both groups.

Atrial strain variables	Dyssynchronous	Synchronous	*p* value
Reservoir LA strain (%)	25.34 ± 8.7	31.30 ± 10.99	0.433
Conduit LA strain (%)	11.62 ± 5.08	15.61 ± 6.99	0.063
Contractile LA strain (%)	14.58 ± 5.8	16.37 ± 5.57	0.963
Strain rate LA reservoir (1/s)	1.16 ± 0.51	1.44 ± 0.58	0.867
Strain rate LA conduit (1/s)	-1.13 ± 0.60	1.63 ± 1.45	0.419
Strain rate LA contraction (1/s)	-1.01 ± 0.61	-1.15 ± 0.66	0.623
Reservoir RA strain (%)	46.81 ± 21.29	52.50 ± 19.13	0.623
Conduit RA strain (%)	20.62 ± 12.59	25.56 ± 14.26	0.787
Contractile RA strain (%)	17.92 ± 13.19	26.73 ± 14.69	0.598
Strain rate RA reservoir (1/s)	1.96 ± 0.83	2.04 ± 0.59	0.336
Strain rate RA conduit (1/s)	-1.52 ± 1.02	-2.10 ± 0.86	0.605
Strain rate RA contraction (1/s)	-1.22 ± 0.74	-1.68 ± 1.31	0.080

Data are expressed as mean and SD. *p* value values for analysis of *t*-test. LA: left atria; RA: right atria.

## Data Availability

Data will be available on request because echocardiography video, electrophysiological records, and confocal images are all extremely big archives.
